# Motor proficiency of learners with moderate to severe intellectual disabilities

**DOI:** 10.4102/ajod.v13i0.1262

**Published:** 2024-02-21

**Authors:** Jose M. Fernandes, Monique de Milander, Elna van der Merwe

**Affiliations:** 1 Independent researcher, Ladybrand, South Africa; 2 Department of Exercise and Sport Sciences, Faculty of Health Sciences, University of the Free State, Bloemfontein, South Africa

**Keywords:** Bruininks–Oseretsky Test of Motor Proficiency, 2nd edition (BOT-2) Brief Form, intellectual disabilities, motor proficiency, motor skill competence

## Abstract

**Background:**

Intellectual disabilities refer to a permanent brain condition that interferes with a learner’s ability to perform basic living tasks, academic tasks and social interactions. By observing the motor proficiency levels of these learners, one can determine the extent of a learner’s possible physical motor proficiency barriers.

**Objective:**

To determine the motor proficiency levels of learners with moderate to severe intellectual disabilities using the Bruininks–Oseretsky Test of Motor Proficiency, second edition (BOT-2) Brief Form.

**Method:**

This quantitative descriptive study included 46 learners (17 girls and 29 boys) from a Mangaung school for learners with special needs between the ages of 15 and 17 years.

**Results:**

Indicated that 31 learners (67.4%) out of 46 learners identified with moderate to severe intellectual disabilities had a well-below average; 11 learners (23.9%) had a below average and only 4 learners (8.7%) had average motor proficiency levels.

**Conclusion:**

Alarmingly, this indicates that the majority of learners have severe motor difficulties that may reduce these learners’ abilities to perform tasks using gross and fine motor skills. Reported motor proficiency levels can be used as a guide to direct future motor intervention programmes.

**Contribution:**

Timely interventions are central to improving learners’ motor difficulties. This study focused on providing information regarding the motor proficiency levels of South African learners with ID that was not previously explored. This was an attempt to bridge the gap in knowledge pertaining to the use of standardised motor proficiency tests for South African learners with ID.

## Introduction

In the American Psychiatric Association (APA 2013), the *Diagnostic and Statistical Manual of Mental Disorders, Fifth Edition* (DSM-V) categorises intellectual disabilities (ID) as having a reduced mental ability in various skills such as reasoning, problem-solving, planning. Furthermore this population struggles to think abstractly, make moral judgements, learn new skills as well as learn from the environment. Intellectual disabilities also cause difficulties in intellectual (conceptual) functioning and social and practical adaptive behaviour (Ashori, Norouzi & Jalil-Abkenar 2018; Schalock et al. 2007). Although learners can either be identified with mild ID, moderate ID, severe ID or profound ID (APA 2013; Barlow et al. 2017; Roth et al. 2017), the focus will mainly be on moderate to severe ID.

Learners with moderate ID usually possess an intelligence quotient (IQ) of between 35–40 and 50–55 (Barlow et al. 2017). These learners have adequate skills to communicate and can perform independent skills with additional support; however, they require assistance in social interactions and decision-making (APA 2013; Roth et al. 2017). Learners with severe ID have a lower IQ score of between 20–25 and 35–40 (Barlow et al. 2017). These learners require extensive support on a daily basis to perform basic self-care skills, constantly need supervision to ensure safety and have rudimentary communication skills (APA 2013; Roth et al. 2017). Didehdar and Kharazinejad (2019) have indicated that the prevalence of ID is rising and affects learners all around the world.

The prevalence of ID is estimated globally, in poor countries and in middle-income countries to be approximately 1.6%, whereas in wealthy countries it is 0.9% (Elmasry, Aladawy & Abd-Elhamid 2020). The literature indicates that males are diagnosed with ID more often than females, with a male:female ratio of 3:2 (Christianson et al. 2002), while other researchers reported a higher ratio of 2.3:1 (Elmasry et al. 2020). While the prevalence of ID is relatively low and affects only a small portion of the population, it is still important to help learners with ID gain a better quality of life and improve their standards of living by improving their motor proficiency levels.

Motor proficiency refers to the ability to perform a wide range of motor skills with competency, including synchronising both fine- and gross motor skills that are essential to perform daily living tasks in order to walk, run, jump, catch, throw, kick and roll (Barnett et al. 2011, 2016; Lopes, Saraiva& Rodriques 2018). In a study investigating the influence of intelligence levels on the motor coordination abilities of intellectually disabled learners, findings reported that the higher the degree of ID, the greater the level of motor proficiency deficits was (Lejcarova 2009). This notation has been evident by several studies (Hartman et al. 2010; Smits-Engelsman & Hill 2012; Vuijk et al. 2010; Westendorp et al. 2011). Fine motor skills involve having control over objects using the arms and hands, that is the smaller muscle groups (Ashori et al. 2018) and incorporate daily skills such as stringing or threading beads, pegging clothes, holding and griping a pencil and a pen in order to copy, trace, draw, paint and to transfer coins (Niechwiej-Szwedo, Alramis & Christian 2017). Gross motor skills involve the large muscles groups (upper and lower extremities) to perform movement actions of jumping, walking, climbing, catching, throwing and striking (Gallahue & Ozmun 2006; Goodway, Ozmun & Gallahue 2021; Loprinzi, Davis & Fu 2015) and being able to use the hands and feet in harmony (Ashori et al. 2018).

Capio, Eguia and Simons (2015) examined the gross motor proficiency by using the Test of Gross Motor Development, second edition (TGMD-2), for 81 learners with ID (65 boys and 16 girls) between the ages of 5 and 14 years. These researchers found that boys performed better in manipulation skills (catching and throwing) than girls. However, findings also stated that none of the learners (boys and girls) could obtain the maximum score of the test, which would demonstrate mastery in motor skills (Capio et al. 2015). Other studies compared the motor proficiency of learners with ID among typically developing learners. Hartman et al. (2010) conducted a study to examine the motor proficiency skills (locomotor and object control skills) and adaptive functioning in learners with borderline ID (*n* = 61) and mild ID (*n* = 36) compared to typically developing learners (*n* = 97). The results of the study revealed that learners with borderline ID and mild ID scored poorer on the TGMD-2 compared to their typically developing peers (Hartman et al. 2010). A study conducted by Rintala and Loovis (2013) examined the motor proficiency skills of 20 learners with mild ID and 20 typical developing learners, aged 7–11 years old, using the TGMD-2. The study revealed that learners with mild ID scored significantly lower in locomotor skills (running, leaping, jumping and sliding) and object control skills (kicking and overhead throwing) compared to typical developing learners (Rintala & Loovis 2013). These aforementioned studies suggest that learners with ID have reduced motor proficiency levels, which makes participation in recreational and sporting activities less likely.

Although most of these studies focused on learners with borderline to moderate ID, it is evident that learners with more severe levels of ID were not well-represented in these studies. Researchers further raised the issue that there is a lack of motor proficiency tests that are properly validated for learners in African countries, to screen for possible motor proficiency deficits (Smits-Engelsman et al. 2022). Moreover, to the best of our knowledge, no studies could be found in South Africa focusing on the motor proficiency levels of learners with moderate to severe ID. Additionally, no research had been conducted on the BOT-2 Brief Form. Therefore, the purpose of the current study was to examine the motor proficiency levels of learners identified with moderate to severe ID (IQ 20–55) using the BOT-2 Brief Form. Improved motor proficiency levels may lead learners with ID in becoming more physically active, reduce sedentary behaviour and allow more successful participation in daily activities.

## Research methods and design

### Study design

The study made use a quantitative descriptive study design to collect data pertaining to the motor proficiency levels of learners identified with moderate to severe ID. Motor proficiency levels were obtained by the primary researcher (a movement specialist) who had received formal training by a qualified Kinderkineticist in the testing procedures of the relevant assessment tool, namely the BOT-2 Brief Form.

### Participants

Participants were recruited from one school for learners with special needs, situated in the Mangaung metropolitan municipality, Free State, South Africa. The process to be admitted to the school was as follows: The Free State Department of Education (DOE) referred learners from mainstream schools because of the fact they have been identified with moderate to severe ID. Intellectual disabilities diagnosis is conducted by a state professional (psychologist employed by the DOE) or medical doctor (either private or state-employed). The school then admits learners according to the criteria set by the DOE with moderate ID, severe ID, profound ID, low-functioning autism, cerebral palsy and Down syndrome. Learners had been diagnosed with an IQ of ≤ 70 by private/public doctors or the relevant state professional. Initially, 120 learners had been included to participate in the study. The schools based their identification on the DOE criteria to identify these learners.

Only learners identified with moderate to severe ID were considered for inclusion in this study. Furthermore, the following exclusion criteria applied: (1) Learners who fell outside the age bracket of 15–17 years or who were not identified with moderate to severe ID; (2) learners who had been diagnosed by a medical physician with any related skeletal disorder, neurological dysfunction, cardiovascular problems or physical disabilities and (3) consent by parents or assent by learners had not been provided.

A total population of 120 learners met the inclusion criteria and consent forms were sent to these parents/guardians. However, only 46 (response rate of 38.3%) agreed to participate in the study. The final study sample included 29 boys (63%) and 17 girls (37%), with a median age of 16 years and 8 months. The youngest participant was 15 years and 6 months and the oldest 17 years and 6 months.

### Procedure

The testing procedure was conducted for a period of 2 weeks during Physical Education and sport periods to prevent learners from missing any formal academic classes. The school screened learners daily for coronavirus disease 2019 (COVID-19) symptoms. Thus, if a learner presented any COVID-19 symptoms, the school referred these learners to the relevant health authorities.

Testing was conducted in line with the national required COVID-19 regulations, and strict adherence was maintained by the primary researcher and the learners. This preventative measure ensured that learners were not potentially exposed to the COVID-19 virus. The primary researcher tested each learner individually in a quiet location and each subtest was laid out according to the guidelines prescribed in the BOT-2 Brief Form manual. The assessment typically took 15–25 min to administer per learner.

The primary researcher divided the learners into chronological age groups consisting of 15-, 16- and 17-year-olds, respectively. All age groups were tested using the same testing procedure. Testing took place in the school hall and upon arrival each learner had to sanitise their hands before starting the test. Furthermore, the primary researcher ensured that the learners’ face mask/face shield was worn properly and that all equipments were sanitised after a learner completed the test. The assessment typically took 15–25 min to administer per learner. The testing areas were set up according to the guidelines prescribed in the BOT-2 test manual and took approximately 10 min (Bruininks & Bruininks 2005). Additionally, 2 min were required to sanitise the equipment and table.

Each learner was assigned a unique number for the duration of the study. The primary researcher fetched the respective learner from the classroom and escorted them to the school hall. Each BOT-2 Brief Form subtest was comprehensively explained to the learner and visually demonstrated. Each learner was allowed a practice round to ensure that an understanding was achieved of what was required of them. Once a learner completed the test, the primary researcher escorted the learner back to the classroom and collected the next learner.

### Measuring instrument

The Bruininks–Oseretsky Test of Motor Proficiency, second edition (BOT-2), uses motor-driven tasks to evaluate the motor proficiency levels of individuals between the ages of 4 and 21 years of age (Bruininks & Bruininks 2010; Cools et al. 2009; Deitz, Kartin & Kopp 2007). It is a standardised assessment tool that can be used to screen the motor proficiency of learners with mild to moderate motor proficiency problems (Bruininks & Bruininks 2010).

The BOT-2 has four administration options, namely the Complete Form, the Short Form, selected composites and the selected subtests (Bruininks & Bruininks 2005). In addition to the above-mentioned options, the Brief Form has recently been made available and has a separate record form, manual and interpretation norms (Bruininks & Bruininks 2010). Motor proficiency is assessed in four main areas: fine manual control, manual coordination, body coordination and agility and strength. These composites can further be divided into the following eight subtests: (1) fine motor precision, (2) fine motor integration, (3) manual dexterity, (4) upper-limb coordination, (5) bilateral coordination, (6) balance, (7) speed and agility and finally (8) strength (Bruininks & Bruininks 2005). The BOT-2 Brief Form was used as it takes less time to administer than the Complete Form. This characteristic is more accommodating to the current study population’s concentration span and abilities. The BOT-2 Brief Form comprises 12 items, where at least one item of each of the mentioned subtests is included (Bruininks & Bruininks 2010).

Raw scores are awarded based on execution of each subtest and can then be converted into a single total point score (Bruininks & Bruininks 2010; Gkotzia, Venetsanou & Kambas 2017). The total point score can be used to calculate standard scores (Cools et al. 2009), percentile values and age equivalents. Using these scores, descriptive categories can be derived for each subtest (if the Complete form is used) and for the total motor proficiency score if the BOT-2 Brief Form/Short Form is used (Bruininks & Bruininks 2005). The five descriptive categories include: well-below average, below-average, average, above-average and well-above average.

The BOT-2 test is a reliable and responsive tool to assess learners with ID motor proficiency levels (Wuang & Su 2009). Furthermore, it has inter-rater reliability of r ≥ 0.90, internal consistency of r ≥ 0.80 and the test–retest reliability of r ≥ 0.80 (Deitz et al. 2007). The construct validity of the BOT-2 test is r = 0.80, while a high correlation (0.80) between the Short Form and the Complete Form has also been reported (Cools et al. 2009).

### Statistical analysis

Descriptive statistics, such as frequencies, percentages for categorical data, means and standard deviations and/or medians, percentiles for numerical data were calculated to determine the motor proficiency levels of learners identified with moderate to severe ID. The principal researcher used a Microsoft Excel 2016 spreadsheet to capture data from the BOT-2 Brief Form electronically. The data were analysed by means of SAS statistical software.

### Ethical considerations

Learners were recruited after consent had been obtained from the Department of Education in the Free State, and from the principal of the school, consenting for the research study to be conducted on the school premises. An application for full ethical approval was submitted to the Health Sciences Research Ethics Committee and ethics consent was received on 07 July 2021. The ethics approval number is UFS-HSD2020/0242/2707. Furthermore, parents or legal guardians had to complete an informed consent document to provide permission for their child to partake in the research study. The learners had to complete the assent form. All documents were written in English, as well as Sesotho (native language) to ensure that parents or legal guardians and learners could have an option to read in a language that they understood. Additionally, the assent forms were designed using picture sentences, so that learners could visually read the assent forms. Participation in the study was completely voluntarily; in addition each participant was given a participant number to maintain the confidentiality of data. The study was overseen in agreement with the Helsinki Declaration as revised in 2013. Learners received food that was provided daily as part of the schools’ national nutritional programme and testing commenced only after they had eaten.

## Results

In Table 1, the descriptive statistics of the total point score, standard score and percentile rank according to the BOT-2 Brief Form results can be observed.

**TABLE 1:** Descriptive statistics of the participants’ motor proficiency levels.

Variable	N	Median	LQ	UQ	MIN	MAX
Total PS	46	42	32	52	2	59
SS	46	26	20	35	20	59
% Rank	46	1	0.9	7	0.9	21

N, sample; LQ, lower quartile; UP, upper quartile; Min, Minimum; Max, Maximum; Total PS, total point score; SS, standard score; %, Percentile.

Results in Table 1 reflect performance on the lower end of possible total point scores, standard scores and percentile ranks, which could be obtained. Minimum values are very low, whereas maximum values reach only average levels.

Table 2 presents the descriptive category, standard score and percentile for the group according to frequency.

**TABLE 2:** Descriptive categories, standard score and percentile rank for the group (N = 46).

Descriptive categories	Standard score			Percentile rank		
	Range	Freq	%	Range	Freq	%
Well-below average	30 or less	31	67.4	2 or less	31	67.4
Below average	31–40	11	23.9	3–17	11	23.9
Average	41–59	4	8.7	18–63	4	8.7

Freq, Frequency.

When interpreting standard score and percentile values, Table 2 indicates that the majority of the learners 67.4% (*n* = 31) were classified as having well-below average motor proficiency abilities. The below average motor proficiency category represented only 23.9% (*n* = 11) of the learners while a very small percentage of learners 8.7% (*n* = 4) obtained average motor proficiency. No learner portrayed motor proficiency abilities at the above average or well-above average levels.

## Discussion

According to our knowledge, this was the first study to establish the motor proficiency abilities of learners characterised with moderate to severe ID, in a special school in the Free State Province of South Africa using the BOT-2 Brief Form. The findings of the current study are in agreement with Bruininks & Bruininks (2010) who discovered that learners with mild to moderate ID had a mean standard score of 26.1. Conversely, we did not use the mean standard score to verify the motor proficiency abilities of learners identified with moderate to severe ID; instead we used the median standard score and established it to be 26. Moreover, it should be noted that the study by Bruininks and Bruininks (2005) examined only learners with mild to moderate ID and did not include any of the severe levels of ID, for instance, moderate to severe ID.

A study led by Wuang, Ho and Su (2013) using the BOT-2 test among learners classified with mild ID (*n* = 73) and fewer learners with moderate ID (*n* = 41) in Taiwan showed results similar to the current study. The baseline of motor ability of learners identified with mild to moderate ID was established. The researchers found that 26.3% of the learners were in category 1 indicating well below average motor proficiency level, 47.4% fell in category 2 representing the below average and 26.3% showed an indication of an average motor proficiency level (Wuang et al. 2013). Similar results were obtained between the studies, indicating than none of the learners were in category 4 and 5, which is above average and average motor proficiency, which proves mastery of the skills (Wuang et al. 2013). A reason that almost half of the participants in the study according to Wuang et al. (2013) fell in the below average motor proficiency level may be ascribed to the sample having more participants with moderate ID, compared to the current study. Additionally, the current study was consistent, as the participants with ID struggled to achieve categories 4 and 5, which is the high end of the motor proficiency spectrum.

Westendorp et al. (2011) conducted research on a Dutch sample and indicated that the learners with mild as well as borderline ID also have impaired gross motor proficiency abilities; specifically, locomotor and manipulation skills associated with typical developing learners. The findings of the current study were consistent with Westendorp et al. (2011). Conversely, we investigated the total motor proficiency that incorporated gross and fine motor activities and established that both these skills were impaired in learners identified with moderate to severe ID. Similar to our research, a Korean sample studied by Jeoung (2018) also indicated that low motor proficiency levels in all areas of development were observed in learners identified with moderate ID. Comparable results were also found in another study conducted in the Netherlands, indicating that learners categorised with mild ID had lower levels of motor proficiency compared to those categorised with borderline ID (Vuijk et al. 2010). A possible explanation for this occurrence is that learners with ID demonstrate deficits in their adaptive functioning (Hartman et al. 2010); thus, suitable performance in complex motor proficiency tasks may be difficult to achieve for learners with ID (Westendorp et al. 2011), especially among those with higher severity levels of ID (Lejcarova 2009).

In South Africa, a recent study was conducted by Smits-Engelsman et al. (2022) and the researcher examined the motor proficiency levels of 6–10 year old typical developing learners. The assessment tool used to measure their abilities was the Movement Assessment Battery for Children second edition (MABC-2) and the BOT-2 Short Form. Approximately a third of the learners from the study were at risk for motor proficiency deficits while using the MABC-2. Furthermore, it is interesting to note that when the BOT-2 Short forms is used, around a tenth of learners were identified with motor proficiency levels deficits (Smit-Engelsman et al. 2022). Even though the current study focused on ID, we found conflicting results, where over two-thirds of the learners had motor proficiency problems.

Smits-Engelsman and Hill (2012) explored the relationship between motor proficiency levels and intelligence across various IQ levels. These researchers established that motor proficiency levels decrease as IQ scores declined or vice versa, indicating that there is a direct connection between intellectual performance and motor proficiency levels (Smits-Engelsman & Hill 2012). Consequently, Vuijk et al. (2010) proposed that it is necessary for schools of special needs to acknowledge that learners’ motor proficiency levels are not a heterogeneous group and a Physical Education session should be adapted to cater for the learners’ existing level of motor proficiency.

Even though some studies have scrutinised the motor proficiency of learners with ID, it becomes apparent that more studies are required to gain an all-inclusive understanding of motor proficiency barriers of these learners, especially for learners with more severe levels of ID. Likewise, the lack of information on the validity of motor proficiency tests for more severe levels of ID, most definitely requires urgent consideration as results cannot be accurately compared. This certainly will cause an issue when selecting an intervention approach to correct possible motor proficiency deficits.

## Conclusion

To our knowledge, this is the first study in South Africa to determine the motor proficiency levels among learners with moderate to severe ID using the BOT-2 Brief Form. The current study indicated that more than two-thirds of the learners categorised with moderate to severe ID fell in the well-below average motor proficiency category. This suggests that motor proficiency delays are present in most of the learners categorised with moderate to severe ID. Deficits in motor proficiency levels can have an adverse impact on a learner’s health, academic progression and participation in sporting activities. Moreover, research regarding the motor proficiency levels on severe levels of ID is limited. Hence, insufficient inferences could be made to the results of the current study, as most of the studies had focused on borderline to moderate ID. Therefore, it is suggested that more information should be obtained for learners with severe levels of ID regarding their motor proficiency levels. Consequently, the results of this study should be made accessible to ensure that the provincial and national departments of education may review the Physical Education curriculum and mediate as soon as possible by employing a motor intervention programme in order to assist these learners.

### Limitations

The current study made use of only one school in the Free State province of South Africa; thus, the results cannot be generalised to other South African special schools. It is therefore recommended that a larger sample be used in the Free State as well as other parts of the country to further inspect the motor proficiency levels of learners identified with moderate to severe ID.

The study also made use of only the age category of 15–17 years. Thus, it is recommended that research be conducted to determine the motor proficiency levels of younger learners identified with moderate to severe ID.

## References

[CIT0001] American Psychiatric Association, 2013, *Diagnostic and statistical manual of mental disorders*, 5th edn., DSM-V, American Psychiatric Association, Arlington, VA.

[CIT0002] Ashori, M., Norouzi, G. & Jalil-Abkenar, S.S., 2018, ‘The effectiveness of motor therapy on motor skills and bilateral coordination of children with intellectual disability’, *Iranian Rehabilitation Journal* 16(4), 331–338. 10.32598/irj.16.4.331

[CIT0003] Barlow, D.H., Durand, V.M., Du Plessis, L. & Visser, C., 2017, *Abnormal psychology: An integrative approach*, Cengage Learning, Andover, viewed 06 May 2021, from https://search.ebscohost.com/login.aspx?direct=trueanddb=nlebkandAN=2281877andsite=ehost-liveandscope=site.

[CIT0004] Barnett, L.M., Lai, S.K., Veldman, S.L.C., Hardy, L.L., Cliff, D.P., Morgan, P.J. et al., 2016, ‘Correlates of gross motor competence in children and adolescents: A systematic review and meta-analysis’, *Sports Medicine* 46, 1663–1688. 10.1007/s40279-016-0495-z 26894274 PMC5055571

[CIT0005] Barnett, L.M., Morgan, P.J., Van Beurden, E., Ball, K. & Lubans, D.R., 2011, ‘A reverse pathway? Actual and perceived skill proficiency and physical activity’, *Medical Science Sports Exercise* 43(5), 898–904. 10.1249/MSS.0b013e3181fdfadd 20962694

[CIT0006] Bruininks, R.H. & Bruininks, B.D., 2005, *Bruininks-Oseretsky test of motor proficiency manual*, 2nd edn., BOT-2, NCS Pearson, Minneapolis, MN.

[CIT0007] Bruininks, R.H. & Bruininks, B.D., 2010, *Bruininks-Oseretsky test of motor proficiency brief form: Manual and administration easel*, 2nd edn., BOT-2 BF, NCS Pearson, Bloomington, MN.

[CIT0008] Capio, C.M., Eguia, K.F. & Simons, J., 2015, ‘Test of gross motor development-2 for Filipino children with intellectual disability: Validity and reliability’, *Journal of Sport Sciences* 34(1), 1–10. 10.1080/02640414.2015.1033643 25888083

[CIT0009] Christianson, A.L., Zwane, M.E., Mange, P., Rosen, E., Venter, A., Downs, D. et al., 2002, ‘Children with intellectual disability in rural South Africa: Prevalence and associated disability’, *Journal of Intellectual Disability Research* 46(2), 179–186. 10.1046/j.1365-2788.2002.00390.x 11869389

[CIT0010] Cools, W., De Martelaer, K., Samaey, C. & Andries, C., 2009, ‘Movement skill assessment of typically developing preschool children: A review of seven movement skill assessment tools’, *Journal of Sport Science and Medicine* 8, 154–168. PMC3761481 24149522

[CIT0011] Deitz, J.C., Kartin, D. & Kopp, K., 2007, ‘Review of the Bruininks-Oseretsky test of motor proficiency, second edition (BOT-2)’, *Physical and Occupational Therapy in Paediatrics* 27(4), 87–102. 10.1300/J006v27n04_06 18032151

[CIT0012] Didehdar, D. & Kharazinejad, A., 2019, ‘The effect of balance activity on down syndrome boys’, *Journal of Physical Health and Sports Medicine* 2, 70–78. 10.36811/jphsm.2019.110012

[CIT0013] Elmasry, H.M.A., Aladawy, M.A-E. & Abd-Elhamid, M.M., 2020, ‘Prevalence and risk factors of intellectual disabilities in children’, *The Egyptian Journal of Hospital Medicine* 81(1), 1307–1313. 10.21608/ejhm.2020.112407

[CIT0014] Gallahue, D.L. & Ozmun, J.C., 2006, *Understanding motor development: Infants, children, adolescents, and adults*, 6th edn., McGraw-Hill, New York, NY.

[CIT0015] Gkotzia, E., Venetsanou, F. & Kambas, A., 2017, ‘Motor proficiency of children with autism spectrum disorders and intellectual disabilities: A review’, *European Psychomotricity Journal* 9(1), 46–49.

[CIT0016] Goodway, J.C., Ozmun, J.C. & Gallahue, D.L., 2021, *Understanding motor development: Infants, children, adolescents, adults*, 8th edn., Jones & Bartlett Learning, Columbus, OH.

[CIT0017] Hartman, E., Houwen, S., Scherder, E. & Visscher, C., 2010, ‚On the relationship between motor performance and executive functioning in children with intellectual disabilities’, *Journal of Intellectual Disability Research* 54(5), 468–477. 10.1111/j.1365-2788.2010.01284.x 20537052

[CIT0018] Jeoung, B., 2018, ‘Motor proficiency differences among students with intellectual disabilities, autism and developmental disability’, *Journal of Exercise Rehabilitation* 14(2), 275–281. 10.12965/jer.1836046.023 29740563 PMC5931165

[CIT0019] Lejcarova, A., 2009, ‘Coordination skills in 9 to 11 years old pupils at practical elementary schools in relationship to their degree of intellectual disability’, *Acta Universitatis Palackianae Olomucensis, Gymnica* 39(4), 53–62, viewed 11 September 2021, from https://gymnica.upol.cz/artkey/gym-2009040006_Coordination_skills_in_9_to_11_years_old_pupils_at_practical_elementary_schools_in_relationship_to_their_degree.php.

[CIT0020] Lopes, V.P., Saraiva, L. & Rodriques, L.P., 2018, ‘Reliability and construct validity of the test of gross motor development-2 in Portuguese children’, *International Journal of Sport and Exercise Psychology* 16(3), 250–260. 10.1080/1612197X.2016.1226923

[CIT0021] Loprinzi, P.D., Davis, R.E. & Fu, Y., 2015, ‘Early motor skill competence as a mediator of child and adult physical activity’, *Preventive Medicine Reports Journal* 2, 833–838. 10.1016/j.pmedr.2015.09.015 PMC4721422 26844157

[CIT0022] Niechwiej-Szwedo, E., Alramis, F. & Christian, L.W., 2017, ‘Association between fine motor skills and binocular visual function in children with reading difficulties’, *Human Movement Science* 56(Part B), 1–10. 10.1016/j.humov.2017.10.014 29096178

[CIT0023] Rintala, P. & Loovis, E.M., 2013, ‘Measuring motor skills in Finnish children with intellectual disabilities’, *Perceptual and Motor Skills: Motor Skills and Ergonomics* 166(1), 294–303. 10.2466/25.10.PMS.116.1.294-303 23829155

[CIT0024] Roth, K., Zittel, L., Pyfer, J. & Auxter, D., 2017, *Principles and methods of adapted physical education and recreation*, 12th edn., Jones and Bartlett Learning, Burlington, MA.

[CIT0025] Schalock, R.L., Luckasson, R.A., Shogren, K.A., Borthwick-Duffy, S., Bradley, V., Buntinx, W.H.E. et al., 2007, ‘The renaming of mental retardation: Understanding the change to the term intellectual disability’, *Intellectual and Developmental Disabilities* 45(2), 116–124. 10.1352/1934-9556(2007)45[116:TROMRU]2.0.CO;2 17428134

[CIT0026] Smits-Engelsman, B. & Hill, E.L., 2012, ‘The relationship between motor coordination and intelligence across the IQ range’, *Pediatrics* 130(4), e950–e956. 10.1542/peds.2011-3712 22987872

[CIT0027] Smits-Engelsman, B., Verbeque, E., Denysschen, M. & Coetzee, D., 2022, ‘Exploring Cultural Bias in Two Different Motor Competence Test Batteries When Used in African Children’, *International Journal of Environmental Research and Public Health* 19, 6788–6800. 10.3390/ijerph19116788 35682371 PMC9180268

[CIT0028] Vuijk, P.J., Hartman, E., Scherder, E. & Visscher, C., 2010, ‘Motor performance of children with mild intellectual disability and borderline intellectual functioning’, *Journal of Intellectual Disability Research* 54(2), 955–965. 10.1111/j.1365-2788.2010.01318.x 20854287

[CIT0029] Westendorp, M., Houwen, S., Hartman, E. & Visscher, C., 2011, ‘Are gross motor skills and sport participation related in children with intellectual disabilities?’, *Research in Developmental Disabilities* 32(3), 1147–1153. 10.1016/j.ridd.2011.01.009 21310587

[CIT0030] Wuang, Y.-P., Ho, G.-S. & Su, C.-Y., 2013, ‘Occupational therapy home program for children with intellectual disabilities: A randomized, controlled trial’, *Research in Developmental Disabilities* 34(1), 528–537. 10.1016/j.ridd.2012.09.008 23085502

[CIT0031] Wuang, Y.-P. & Su, C.-Y., 2009, ‘Reliability and responsiveness of the Bruininks-Oseretsky Test of motor proficiency-second edition in children with intellectual disability’, *Research in Developmental Disabilities* 30(5), 847–855. 10.1016/j.ridd.2008.12.002 19181480

